# Supplementary material for: Plastoquinone homoeostasis by Arabidopsis proton gradient regulation 6 is essential for photosynthetic efficiency
Source: Commun Biol. 2019 Jun 20;2:220. doi: 10.1038/s42003-019-0477-4 (PMC6586890; doi:10.1038/s42003-019-0477-4)
Supplement: Supplementary file 2 — Supplementary Information [file 42003_2019_477_MOESM2_ESM.pdf]

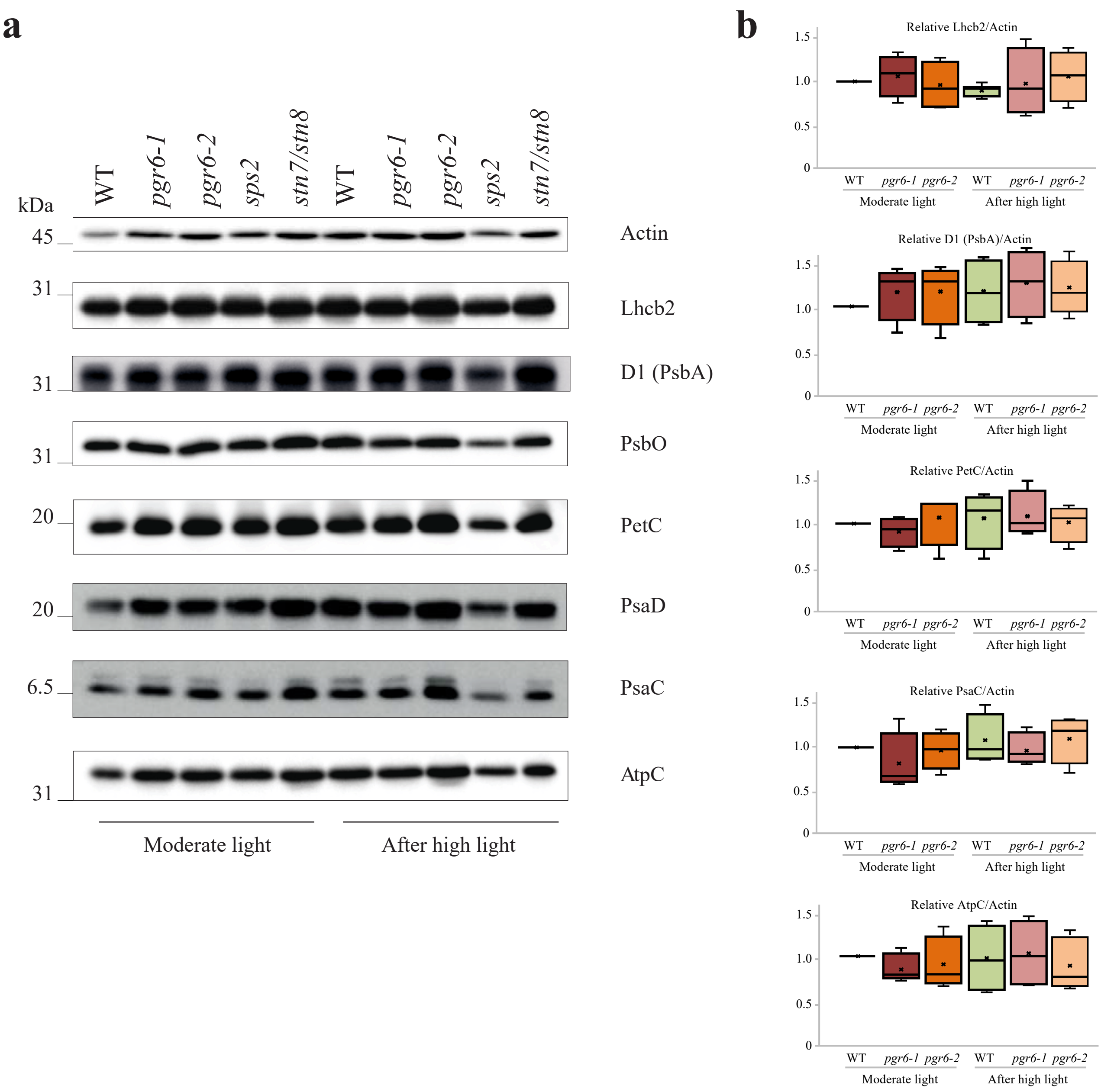

Supplementary Figure 1. Abundance of photosynthetic complexes is not affected in *pgr6* mutants. Total protein extract of light-exposed leaves under moderate light (120 µmol·m^{-2}·s^{-1}) and after 3h of high light (500 µmol·m^{-2}·s^{-1}) from wild type (WT), *pgr6-1*, *pgr6-2*, *sps2* and *stn7/stn8* adult plants were separated in SDS-PAGE. **(a)** Upon transfer on nitrocellulose membrane, representative subunits of the principal photosynthetic complexes were immuno-detected. Lhcb2 for the major LHCII, D1 (PsbA) and PsbO for PSII, PetC for cytochrome *b6f*, PsaD and PsaC for PSI, and AtpC for ATP synthase. Actin was used as a loading control. Uncropped images of the membranes used for immuno-detection are available in Supplementary Fig. 10. **(b)** For WT as well as *pgr6-1* and *-2* representative proteins for each photosynthetic complex were quantified using gel image analysis software (ImageQuant GE healthcare) and normalized to actin, the graph shows the result, whiskers and box plot shows the minimum, first quartile, median, average, third quartile, and maximum of each data set (n=4 biologically independent samples). Data points for the item b are available in Supplementary data 8.

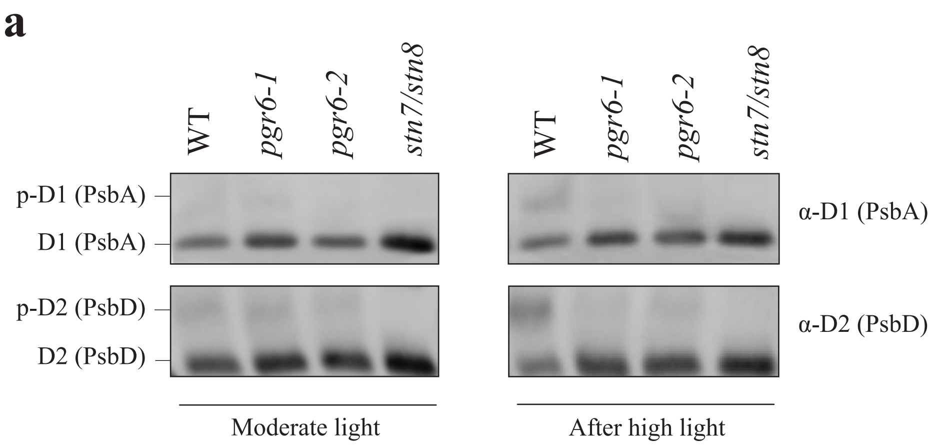

b

	WT	*pgr6-1*	*pgr6-2*	*stn7/stn8*
Fo (ML)	41.32 ± 1.34	43.86 ± 1.79	45.02 ± 1.82	48.10 ± 2.83
Fo (HL)	39.55 ± 2.07	41.27 ± 2.72	43.46 ± 1.63	43.18 ± 1.12

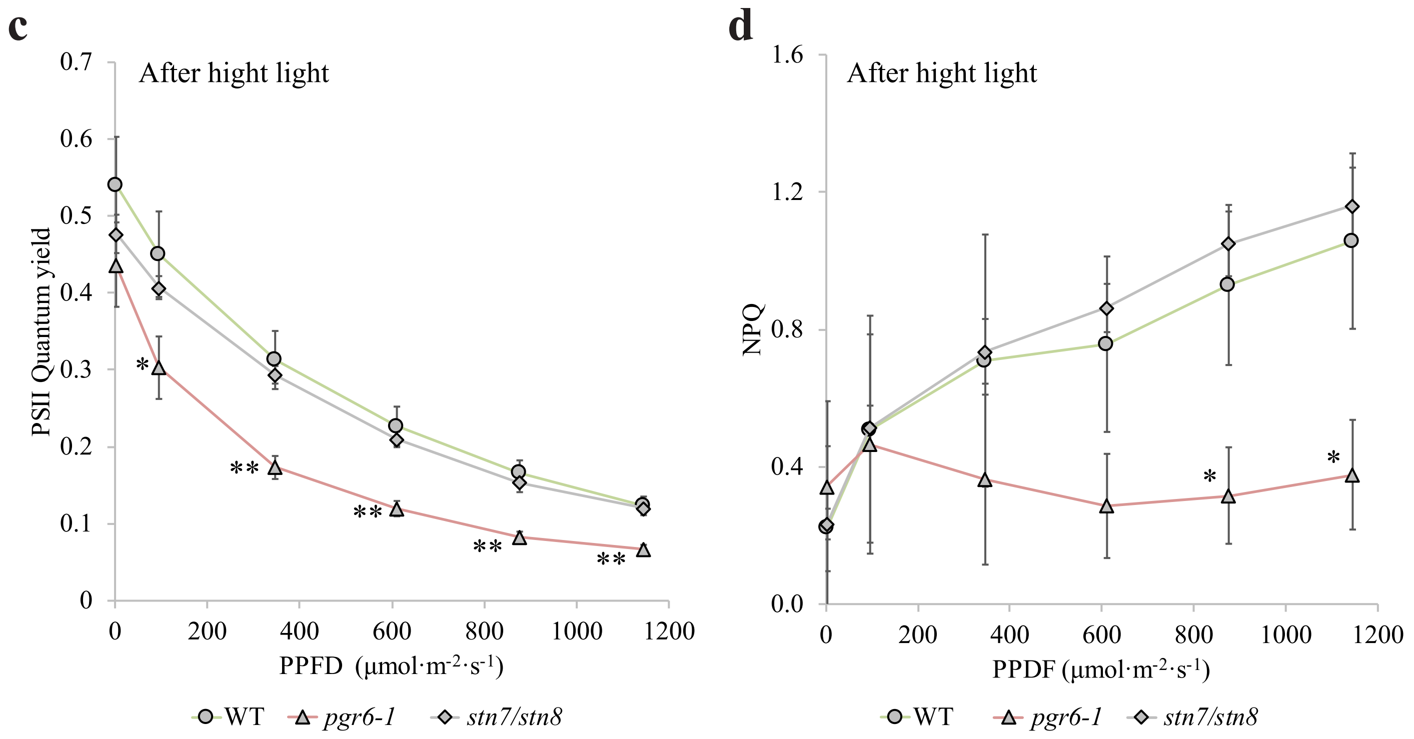

Supplementary Figure 2. Reduced phosphorylation of thylakoid proteins does not strongly affect PSII functionality in *pgr6* mutants after short high light treatment. Total proteins of wild type (WT), *pgr6-1*, *pgr6-2* and *stn7/stn8* adult plants before (Moderate light) and after high light treatment were separated on Phostag-pendant acrylamide gels. **(a)** D1 (PsbA) and D2 (PsbD) phosphorylation level visualised using D1 (PsbA) and D2 (PsbD) antibodies, the upper band corresponds to the phosphorylated form (p-), a protein sample from *stn7/stn8* double mutant was loaded as a non-phosphorylated control. Uncropped images of the membranes used for immuno-detection are available in Supplementary Fig. 12. **(b)** Average F0 values measured after 10 minutes of dark relaxation in wild type (WT), *pgr6-1*, *pgr6-2* and *stn7/stn8*. The plants were kept under moderate light (120 µmol·m^{-2}·s^{-1}) (ML) or under high light (500 µmol·m^{-2}·s^{-1}) for 3h (HL). PSII quantum yield **(c)** and the NPQ **(d)** measured in plants exposed to 3h of HL for WT, *pgr6-1* and *stn7/stn8*. The measurements were performed with Fluorcam (MF800 – PSI). Each value represents the average of a pot containing 2-3 plants. Error bars indicate ±SD between different pots (n=3). (Student's t-test, ** : p<0.01; * : p<0.05). Data points for the items b, c and d are available in Supplementary data 1.

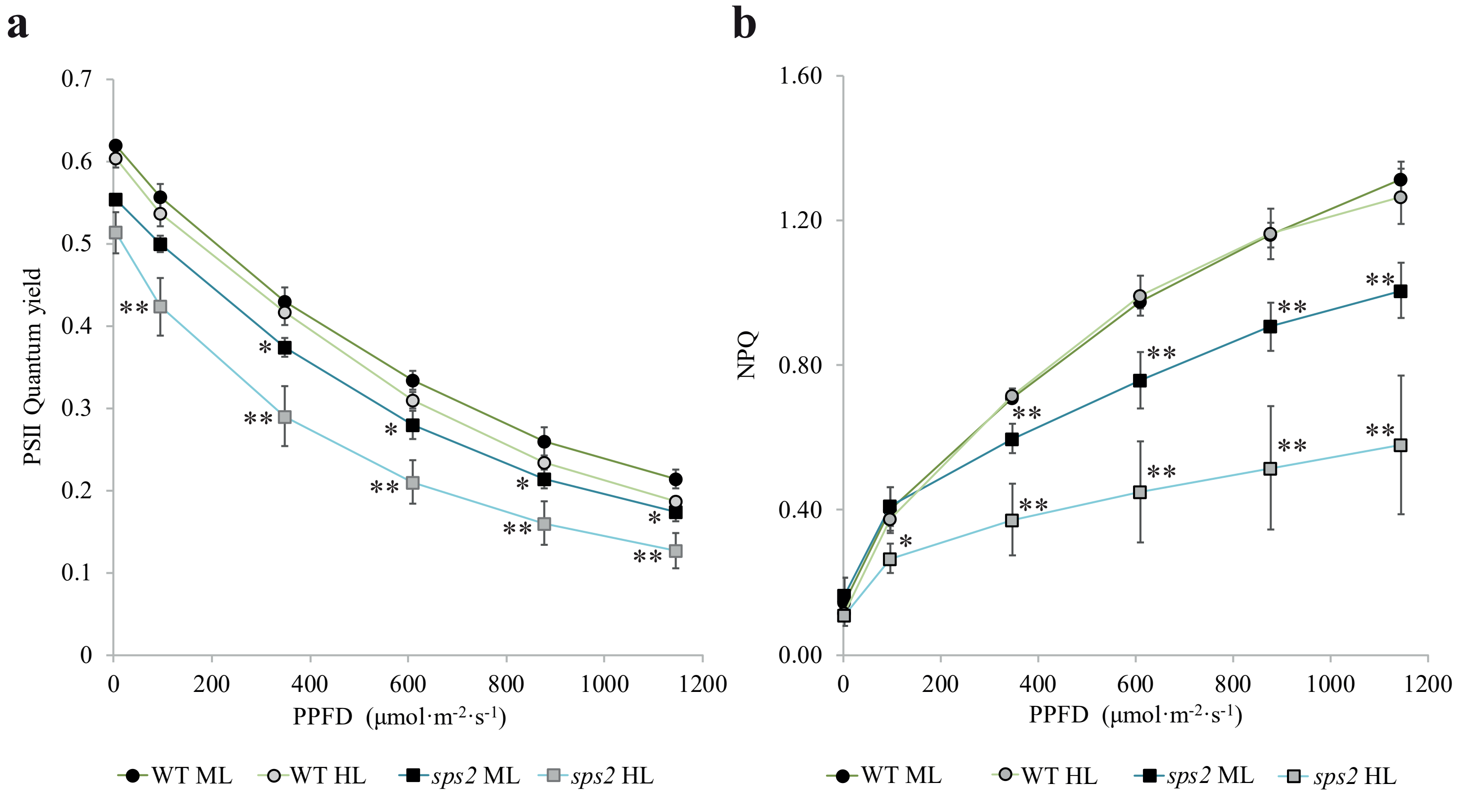

Supplementary Figure 3. Mutant of PQ biosynthesis (*sps2*) is affected in photosystem II quantum yield and thermal dissipation. 24 days old plants grown on soil in short day cycle (8h light /16h dark) were used to assess the photosynthetic efficiency of wild type (WT) and *sps2* under moderate light (120 µmol·m^{-2}·s^{-1}) (ML, dark colors) and after 3 hours of high light (500 µmol·m^{-2}·s^{-1}) (HL, light colors). After 10 minutes of dark relaxation variable room temperature chlorophyll fluorescence was measured on whole plants exposed to these light conditions to determine the following parameters: **(a)** PSII quantum yield (ΦPSII = (FM' – FS)/FM') at increasing light intensities and **(b)** non-photochemical quenching (NPQ = (FM – FM')/FM') after one minute of exposure at different light intensities. These measures were performed with a Fluorcam (MF800 – PSI) with blue light LEDs (470 nm). Each value represents the average of a pot containing 2-3 plants. Error bars indicate ±SD between different pots (n=3). Asterisks indicate statistically different points in which the difference between *sps2* and the wild type exposed to the same light treatment (Student's t-test, ** : $p<0.01$; * : $p<0.05$). Data points for the items a and b are available in Supplementary data 1.

a

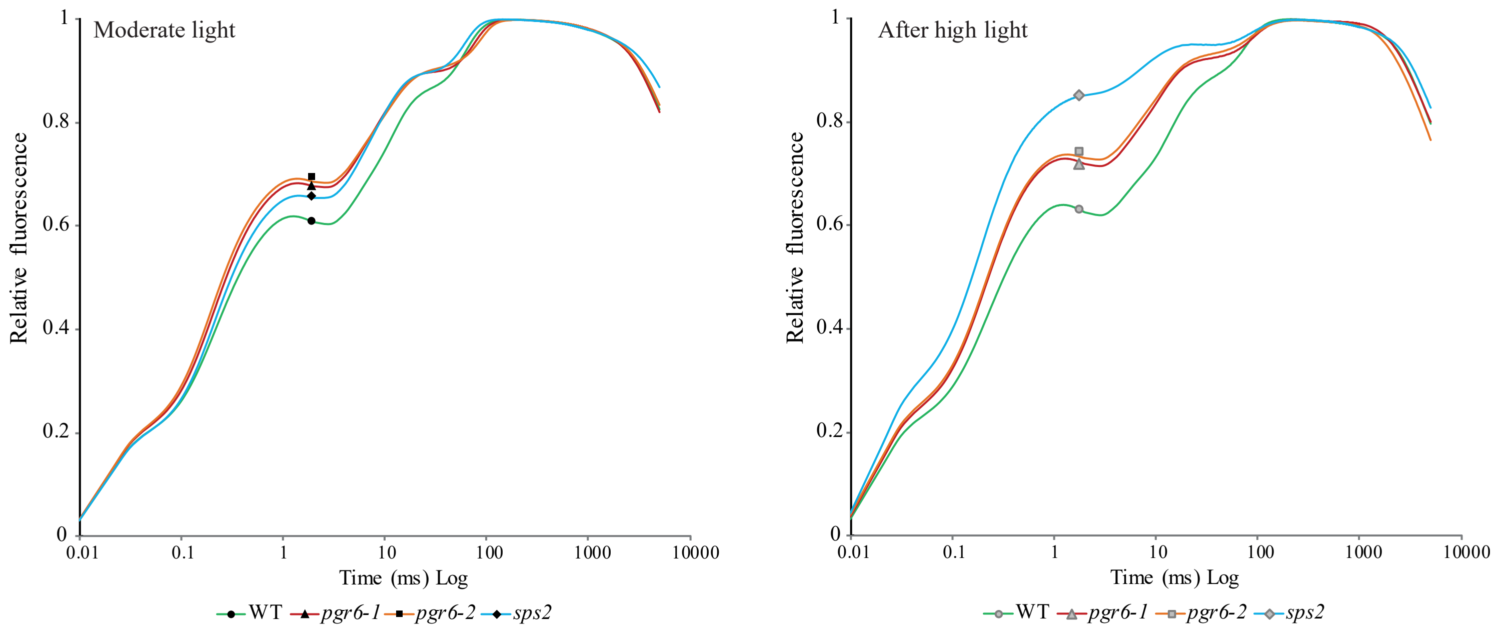

b

**Moderate light**	WT	*pgr6-1*	*pgr6-2*	*sps2*
ΦPo	0.97±0.00	0.97±0.00	0.97±0.00	0.97±0.00
ΦET2o	0.40±0.05	**0.35±0.06****	**0.33±0.07****	**0.34±0.05****
ΦRE1o	0.13±0.02	**0.11±0.03***	**0.11±0.01****	**0.10±0.01****

**After high light**	WT	*pgr6-1*	*pgr6-2*	*sps2*
ΦPo	0.97±0.00	0.96±0.00	0.96±0.02	0.96±0.00
ΦET2o	0.38±0.03	**0.28±0.04****	**0.28±0.07****	**0.15±0.05****
ΦRE1o	0.13±0.02	**0.08±0.02****	**0.07±0.03****	**0.05±0.02****

Supplementary Figure 4. Analysis of electron flux impairment in *pgr6* mutants by rapid fluorescence induction. (a) Average traces of rapid chlorophyll *a* fluorescence induction in logarithmic scale measured using a Plant Efficiency Analyser (Handy-PEA Hansatech Instruments). Biologically independent adult leave samples from wild type (WT) (n=8), *pgr6-1* (n=6), *pgr6-2* (n=8), *sps2* (n=4) harvested under moderate light (120 $\mu mol \cdot m^{-2} \cdot s^{-1}$) (left) and after 3 hours of high light (500 $\mu mol \cdot m^{-2} \cdot s^{-1}$) (right). **(b)** Calculated electron fluxes from the induction curve using the JIP-test as described in Strasser *et al.* (2010)[1] and Kalaji *et al.* (2014)[2]. ΦPo (maximum quantum yield of primary PSII photochemistry), ΦET2o (quantum yield of the electron transport from Q_A to Q_B) and ΦRE1o (quantum yield of the electron transport until the PSI electron acceptors). The mean and the standard deviation is reported, values statistically different from the wild type are shown in bold (Student's t-test,** $p<0.05$; * $p<0.10$). Data points for the items a and b are available in Supplementary data 5.

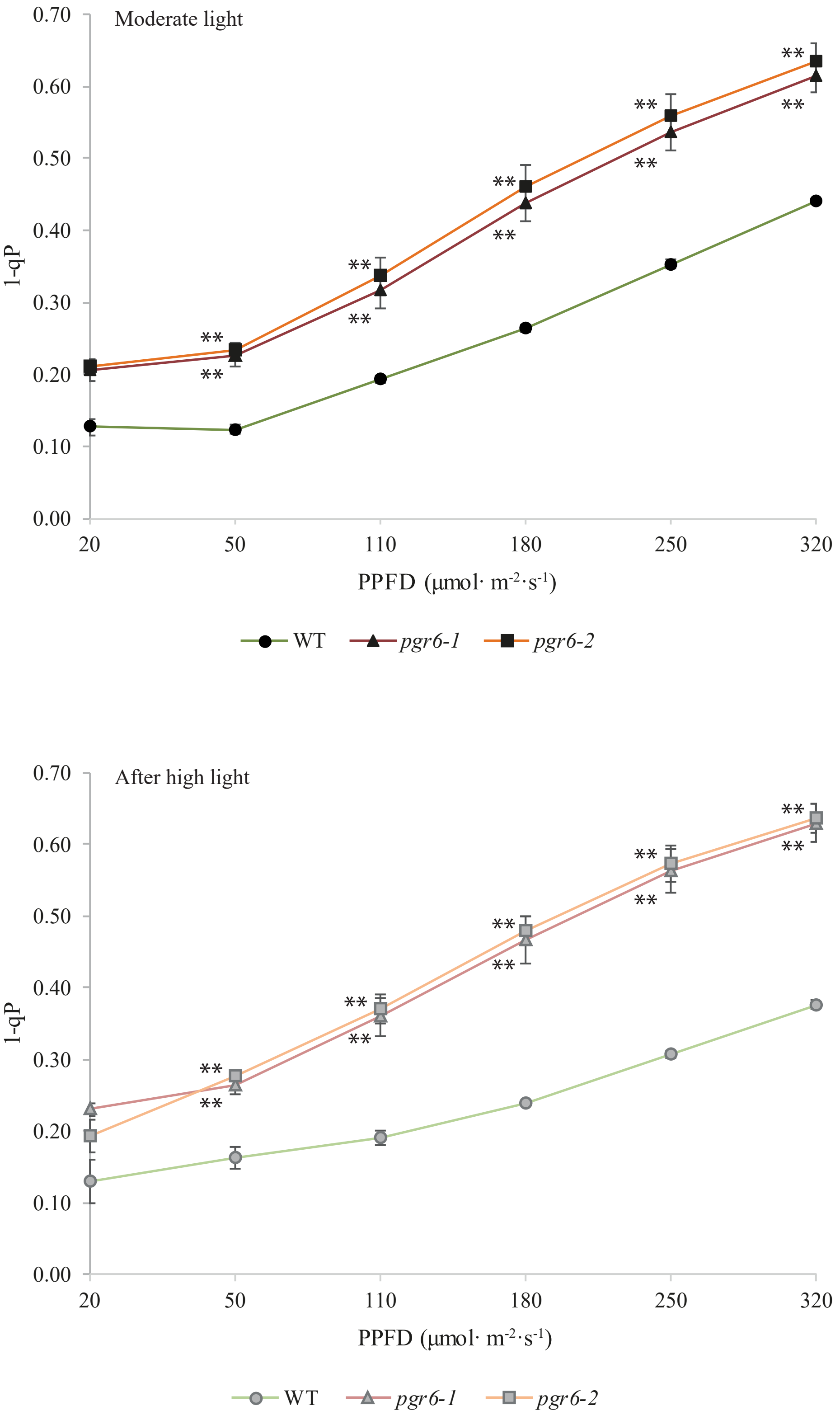

Supplementary Figure 5. The fraction of "closed" PSII reaction centres increases in *pgr6* mutants. The fraction of closed Q_A sites, expressed as 1-qP, was calculated from room temperature chlorophyll fluorescence in wild type (WT), *pgr6-1* and *pgr6-2* adult plants exposed to moderate light (120 µmol·m^{-2}·s^{-1}) and after 3h of high light (500 µmol·m^{-2}·s^{-1}). Each value represents the average of a pot containing 2-3 plants. Error bars indicate ±SD between different pots (n=3). (Student's t-test, ** : $p<0.01$). These measurements were performed with a Fluorcam (MF800 – PSI) with red light LEDs (660 nm). Data points for the items are available in Supplementary data 1.

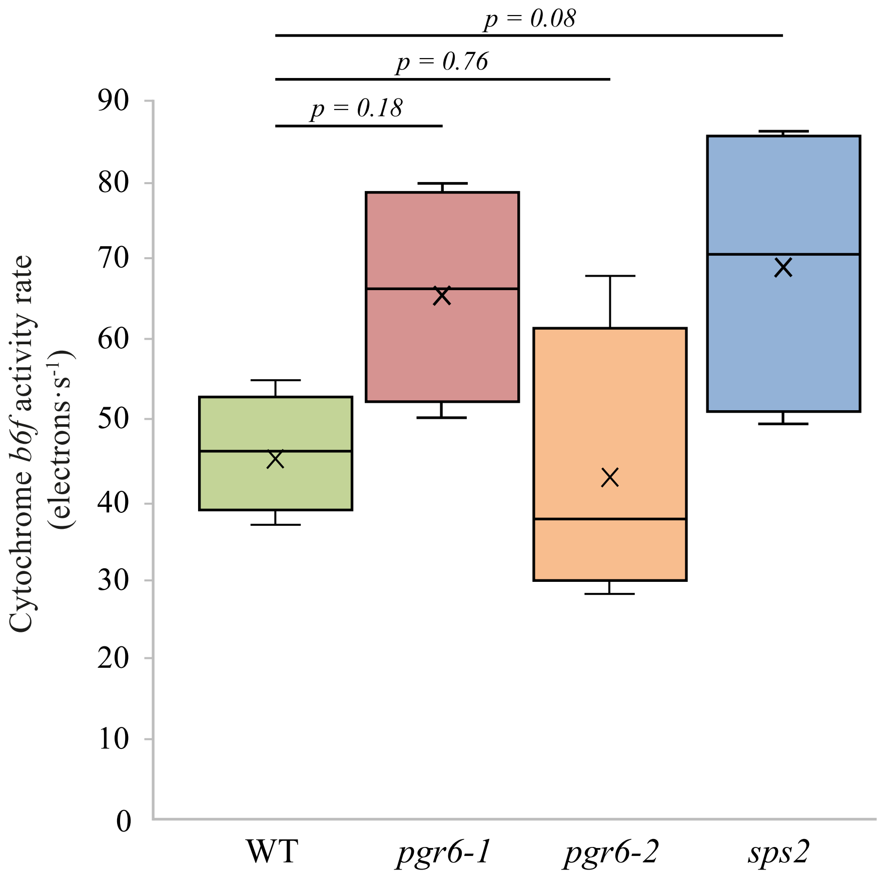

Supplementary Figure 6. High light treatment does not perturb the cytochrome *b6f* turnover in *pgr6* mutant. Wild type (WT) (n=4), *pgr6-1* (n=6), *pgr6-2* (n=4) and *sps2* (n=4) independent plants were grown under moderate light condition, fully expanded leaves were collected after 3h of high light (500 µmol·m^{-2}·s^{-1}). The turnover rate of the cytochrome *b6f* (expressed as electrons per second) was calculated by fitting the kinetics of the cytochrome *f* oxidation after a saturating pulse with an exponential curve. The measured signal was the absorption at 554 nm corresponding to the cytochrome *f* as described in Finazzi *et al.* (2002)[3]. Whiskers and box plot shows the minimum, first quartile, median, average, third quartile, and maximum of each data set. Mutant lines were compared to wild type via a Student's t-test and corresponding p-values reported above each one. Data points are available in Supplementary data 7.

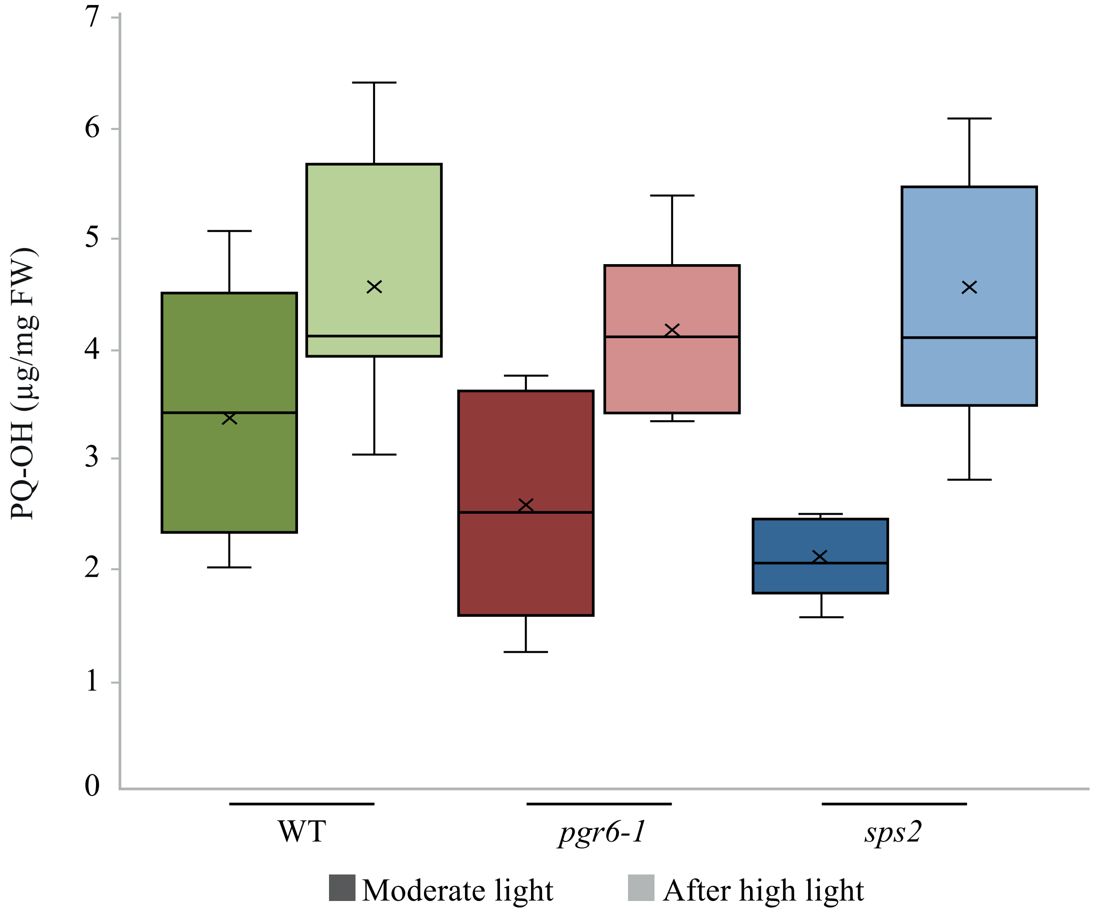

Supplementary Figure 7. Hydroxyl-plastoquinone accumulation in leaves. Hydroxyl-plastoquinone (PQ-OH) accumulation in total rosette from wild type (WT) (n=7), *pgr6-1* (n=5) and *sps2* (n=5) independent plants exposed to moderate light (120 µmol·m^{-2}·s^{-1}) and 3h of high light (500 µmol·m^{-2}·s^{-1}). Identification and quantification of this molecule performed as described in Eugeni-Piller *et al.* (2014)[4] and in Martinis *et al.* (2011)[5]. Whiskers and box plot shows the minimum, first quartile, median, average, third quartile, and maximum of each data set, n values represent the number of independent plants analysed for each light condition. Data points are available in Supplementary data 6.

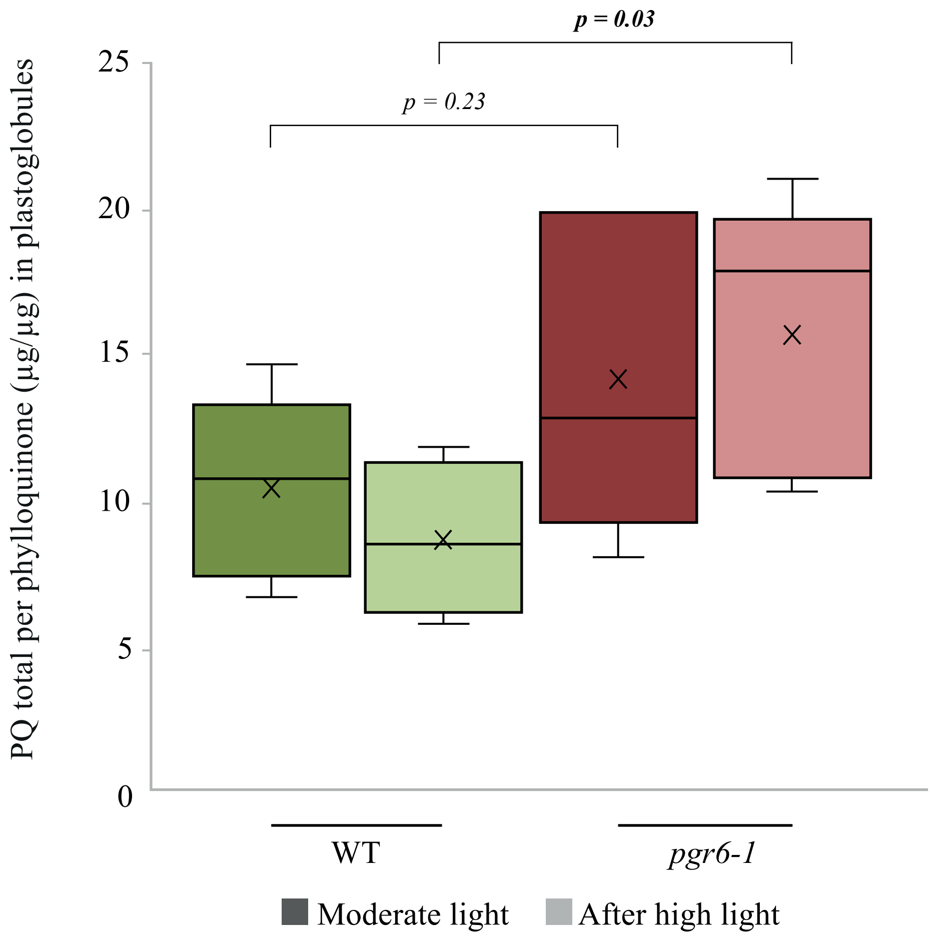

Supplementary Figure 8. Plastoquinone accumulation in plastoglobules. Total plastoquinone (PQ) of wild type (WT) and *pgr6-1* plastoglobules fractions prepared from plants exposed to moderate light (120 µmol·m^{-2}·s^{-1}) and after 3h of high light (500 µmol·m^{-2}·s^{-1}). Plastoglobules were isolated by flotation on a sucrose gradient and prenyl lipids were analysed as described in Eugeni-Piller *et al.* (2014)[4] and in Martinis *et al.* (2011)[5]. Total plastoquinone content (oxidised + reduced PQ) was normalised on phylloquinone (µg/µg). Whiskers and box plot shows the minimum, first quartile, median, average, third quartile, and maximum of each data set (n=5 biologically independent samples). Wild type and *pgr6-1* were compared by Student's t-test; corresponding p-values are reported above each compared group. Data points are available in Supplementary data 6.

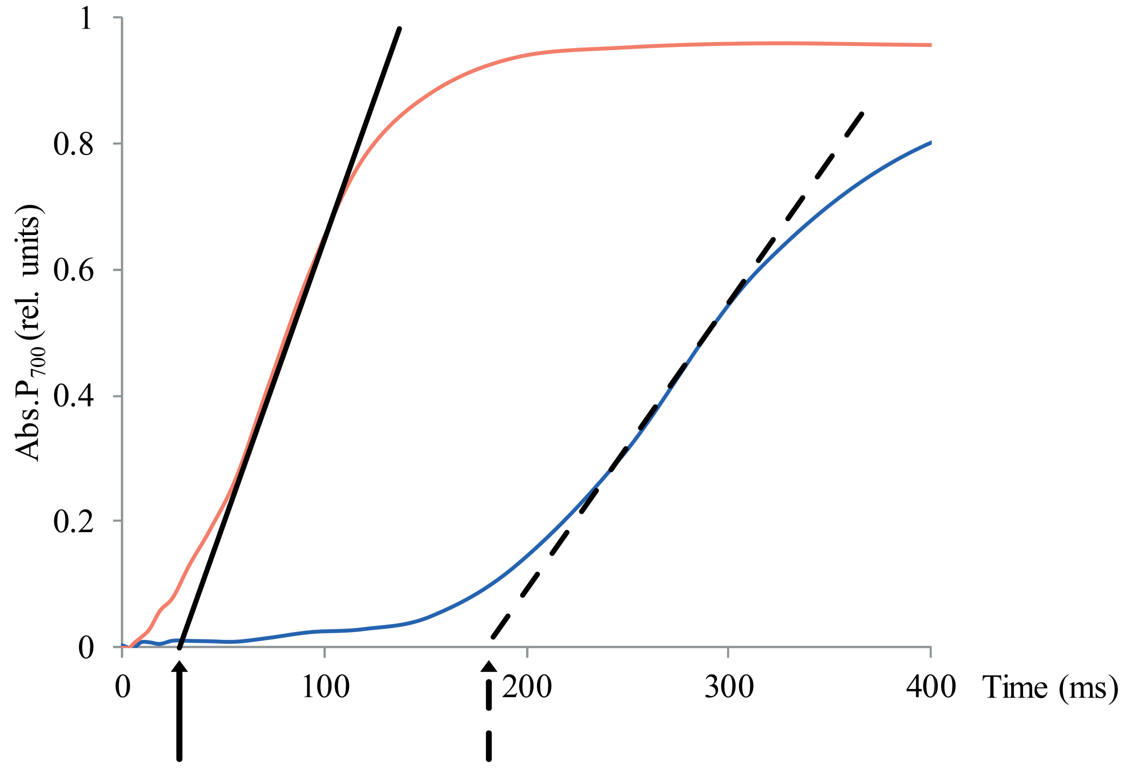

Supplementary Figure 9. Calculation of the ETC capacity to maintain a reduced P_{700}. The lag time from the start of far-red illumination (T0) to the P_{700} oxidation event was calculated in the presence of the light pulse (PSI electron donors reduced) (blue line) or in the absence of the pulse (PSI donors oxidised) (orange line). The lag value was inferred from the intersection between the linear function interpolating the slope and the "x" axis for both conditions (solid line, lag without the saturating pulse; dashed line, lag measured after the saturating pulse). The ratio between these two values is used as a proxy for the number of available electrons per PSI.

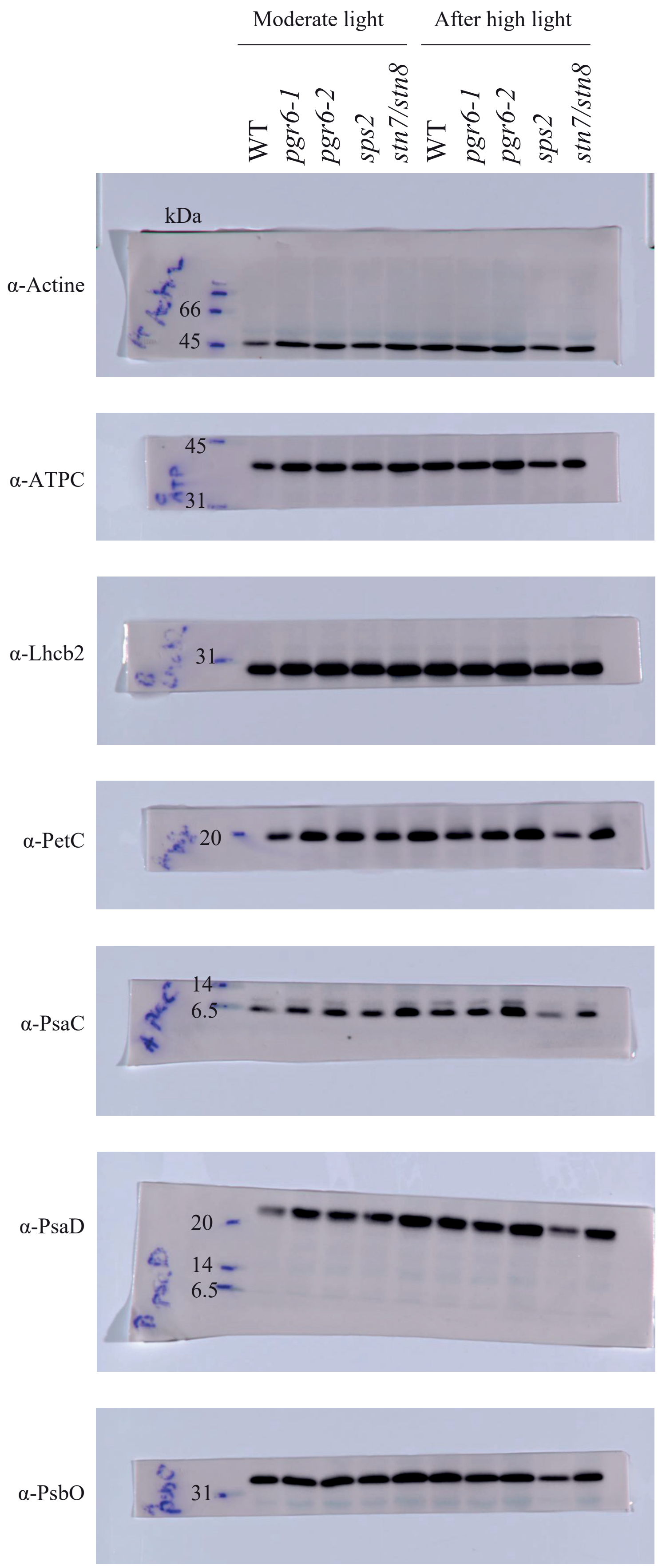

Supplementary Figure 10. Uncropped membrane images. Uncropped images of the membranes used for the immunodetection shown in supplementary figure 1, membranes were cut before the immunodetection.

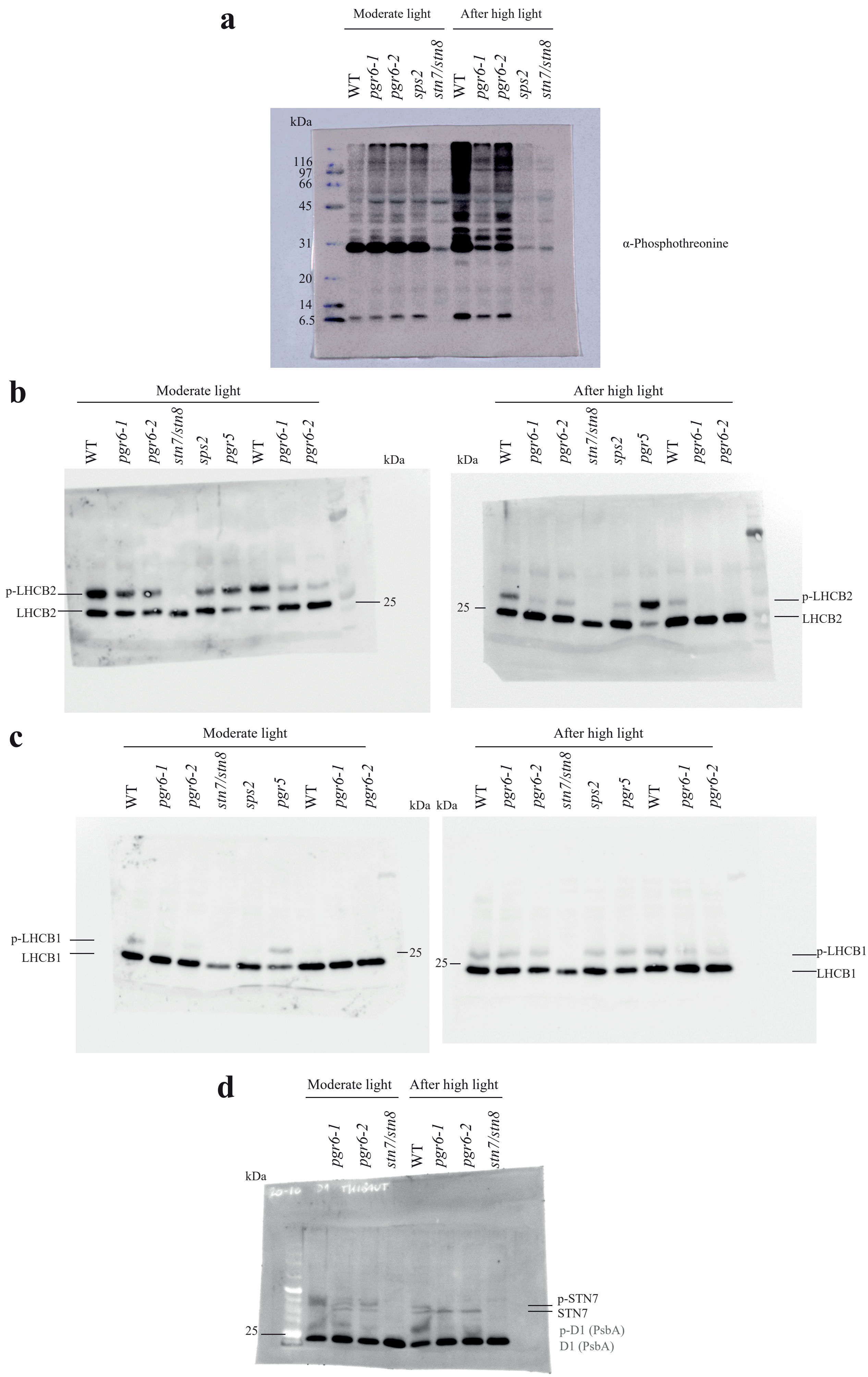

Supplementary Figure 11. Uncropped membrane images. Uncropped images of the membranes used for the immunodetection shown in figure 2 and supplementary figure 1. In **b**,**c** and d the position of the 25 KDa band is shown (Color Prestained Protein Standard, Broad Range (11–245 kDa), NEB), please note that in phostag containing gel the protein migration is not completely correspondent to the molecular weight. For **d** please note that the anti-STN7 antibody was probed on the membrane used for D1 (PsbA) detection, therefore the previous signal is still visible on this membrane.

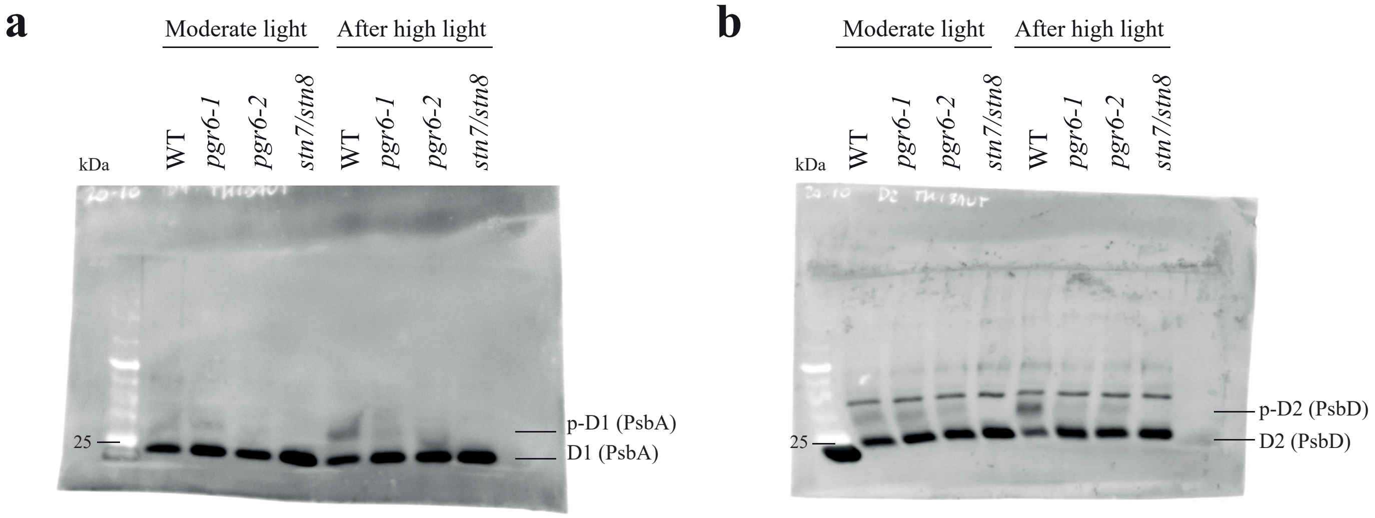

Supplementary Figure 12. Uncropped membrane images. Uncropped images of the membranes used for the immunodetection shown supplementary figure 2. The position of the 25 KDa band is shown (Color Prestained Protein Standard, Broad Range (11–245 kDa), NEB), please note that in phostag containing gel the protein migration is not completely correspondent to the molecular weight.

References

1 Strasser, R. J., Tsimilli-Michael, M., Qiang, S. & Goltsev, V. Simultaneous in vivo recording of prompt and delayed fluorescence and 820-nm reflection changes during drying and after rehydration of the resurrection plant Haberlea rhodopensis. *Biochim Biophys Acta* **1797**, 1313-1326, doi:10.1016/j.bbabio.2010.03.008 (2010).

2 Kalaji, H. M., Oukarroum, A., Alexandrov, V., Kouzmanova, M., Brestic, M., Zivcak, M., Samborska, I. A., Cetner, M. D., Allakhverdiev, S. I. & Goltsev, V. Identification of nutrient deficiency in maize and tomato plants by in vivo chlorophyll a fluorescence measurements. *Plant Physiol Biochem* **81**, 16-25, doi:10.1016/j.plaphy.2014.03.029 (2014).

3 Finazzi, G., Rappaport, F., Furia, A., Fleischmann, M., Rochaix, J. D., Zito, F. & Forti, G. Involvement of state transitions in the switch between linear and cyclic electron flow in Chlamydomonas reinhardtii. *EMBO Rep* **3**, 280-285, doi:10.1093/embo-reports/kvf047 (2002).

4 Eugeni-Piller, L., Glauser, G., Kessler, F. & Besagni, C. Role of plastoglobules in metabolite repair in the tocopherol redox cycle. *Front Plant Sci* **5**, doi:10.3389/Fpls.2014.00298 (2014).

5 Martinis, J., Kessler, F. & Glauser, G. A novel method for prenylquinone profiling in plant tissues by ultra-high pressure liquid chromatography-mass spectrometry. *Plant Methods* **7**, 23, doi:10.1186/1746-4811-7-23 (2011).
