# Supplementary material for: Plastoquinone homoeostasis by Arabidopsis proton gradient regulation 6 is essential for photosynthetic efficiency
Source: Commun Biol. 2019 Jun 20;2:220. doi: 10.1038/s42003-019-0477-4 (PMC6586890; doi:10.1038/s42003-019-0477-4)
Supplement: Supplementary file 3 — Description of Additional Supplementary Files [file 42003_2019_477_MOESM3_ESM.docx]

**Description of additional supplementary items**

**Supplementary Data 1.** Data used for the photosynthetic parameters displayed in figure 1.

**Supplementary Data 2.** Raw fluorescence data and elaborated parameters displayed in figure 2.

**Supplementary Data 3.** Data used for the graph showed in figure 3a.

**Supplementary Data 4.** Raw absorption data and elaborated parameters displayed in figure 3b and Table 1.

**Supplementary Data 5.** Raw fluorescence data and elaborated parameters displayed in supplementary figure 4.

**Supplementary Data 6.** Measured concentration of plastoquinone as displayed in figure 3c and d.

**Supplementary Data 7.** Measured values of the cytochrome *b_6_f* turnover rate displayed in supplementary figure 5.

**Supplementary Data 8.** Quantification data from protein immunodetection displayed in supplementary figure 1
